# Rapid Triage of Children with Suspected COVID-19 Using Laboratory-Based Machine-Learning Algorithms

**DOI:** 10.3390/v15071522

**Published:** 2023-07-08

**Authors:** Dejan Dobrijević, Gordana Vilotijević-Dautović, Jasmina Katanić, Mirjana Horvat, Zoltan Horvat, Kristian Pastor

**Affiliations:** 1 Faculty of Medicine, University of Novi Sad, 21000 Novi Sad, Serbia; gordana.vilotijevic-dautovic@mf.uns.ac.rs (G.V.-D.); jasmina.katanic@mf.uns.ac.rs (J.K.); 2 Institute for Child and Youth Health Care of Vojvodina, 21000 Novi Sad, Serbia; 3 Faculty of Civil Engineering Subotica, University of Novi Sad, 24000 Subotica, Serbia; isicm@gf.uns.ac.rs (M.H.); horvatz@gf.uns.ac.rs (Z.H.); 4 Faculty of Technology, University of Novi Sad, 21000 Novi Sad, Serbia; kristian.pastor@uns.ac.rs

**Keywords:** children, infection, COVID-19, machine learning, laboratory

## Abstract

In order to limit the spread of the novel betacoronavirus (SARS-CoV-2), it is necessary to detect positive cases as soon as possible and isolate them. For this purpose, machine-learning algorithms, as a field of artificial intelligence, have been recognized as a promising tool. The aim of this study was to assess the utility of the most common machine-learning algorithms in the rapid triage of children with suspected COVID-19 using easily accessible and inexpensive laboratory parameters. A cross-sectional study was conducted on 566 children treated for respiratory diseases: 280 children with PCR-confirmed SARS-CoV-2 infection and 286 children with respiratory symptoms who were SARS-CoV-2 PCR-negative (control group). Six machine-learning algorithms, based on the blood laboratory data, were tested: random forest, support vector machine, linear discriminant analysis, artificial neural network, k-nearest neighbors, and decision tree. The training set was validated through stratified cross-validation, while the performance of each algorithm was confirmed by an independent test set. Random forest and support vector machine models demonstrated the highest accuracy of 85% and 82.1%, respectively. The models demonstrated better sensitivity than specificity and better negative predictive value than positive predictive value. The F1 score was higher for the random forest than for the support vector machine model, 85.2% and 82.3%, respectively. This study might have significant clinical applications, helping healthcare providers identify children with COVID-19 in the early stage, prior to PCR and/or antigen testing. Additionally, machine-learning algorithms could improve overall testing efficiency with no extra costs for the healthcare facility.

## 1. Introduction

The coronavirus disease (COVID-19), caused by a novel strain of betacoronavirus (SARS-CoV-2), has marked the past two years with over 6.7 million deaths worldwide. New cases are still being diagnosed but with predominantly mild clinical manifestations. COVID-19 primarily occurs in the adult population, but children play a significant role in the spread of the disease. Children with COVID-19 usually only have a few mild symptoms or no symptoms at all, which is why they remain unrecognized. Additionally, newborns, infants, and toddlers cannot wear protective masks in an appropriate way, and moreover, they cannot clearly describe their health condition. Regardless of the disease’s severity and the amount of viral load, it is important to consider that pediatric patients may contribute to the transmission chain. For all of the above reasons, the pediatric population should receive special attention during the current pandemic [1,2,3].

Even though viral pneumonia has been recognized as the main clinical presentation of this disease, representing the main cause of its severity and mortality, SARS-CoV-2 infection may cause several complications in other organs, such as coagulation disorders (pulmonary embolism, venous thromboembolism, hemorrhages, and acute ischemic stroke) with abdominal involvement (acute mesenteric ischemia, pancreatitis, and acute kidney injury), especially in severely ill patients and those admitted to the ICU, even in children [4,5]. According to the available data, fever occurs in almost 90% of patients and weakness in 70% of patients. A dry cough is present in more than 60% of patients. Nausea and vomiting are pronounced in 5% of patients, and diarrhea occurs in almost 4% of patients [6]. Guan et al. [7] showed that 15.74% of the patients had a severe clinical form of the disease. During hospital treatment, more than 90% of patients were diagnosed with pneumonia. Acute respiratory distress syndrome (ARDS) was confirmed in 3.4% of patients and septic shock in slightly more than 1% of patients. Comorbid diseases, such as hypertension, cardiomyopathy, coronary artery disease, chronic kidney diseases, chronic lung disease, etc., are significantly more common in patients with severe symptoms of the disease (38%) compared to those with a milder form of the disease (21%). Based on the laboratory findings, the occurrence of lymphopenia, which is commonly observed in adults with COVID-19, was found in laboratory tests in only 5.5% of children diagnosed with the disease. The estimated prevalence of leukopenia in pediatric COVID-19 patients was found to be 7.3%. The prevalence rates for high C-reactive protein (CRP) levels, high LDH levels, high creatine kinase MB (CK-MB) levels, high AST levels, and high erythrocyte sedimentation rate (ESR) were estimated to be 14.0%, 17.4%, 43%, 12.3%, and 29.7%, respectively [8]. SARS-CoV-2 infection was commonly followed by hyperinflammation due to the excessive production of proinflammatory cytokines, such as IL-1, IL-2, IL-6, IL-15, IL-18, TNF-α, IFN-γ, etc. Numerous cytokines have been tested in order to reduce mortality, especially in critically ill patients [9].

The gold standard for confirming the presence of the viral genome in a biological sample is quantitative polymerase chain reaction (qPCR). Chest X-rays and computed tomography (CT) were regarded as the main diagnostic tools for the diagnosis of COVID-19. However, given the progressively increased availability of RT-PCR, CT changed from being primarily a diagnostic tool to playing a prognostic role. In fact, evaluation of the CT score became essential for proper patient management, proving to be essential for deciding whether to hospitalize the patient in healthcare settings with limited resources and a shortage of intensive care beds [10]. Although qPCR is an irreplaceable diagnostic tool in the current pandemic, a prolonged turnaround time is often a significant issue. Additionally, molecular diagnostics is relatively expensive, especially for developing countries, and represents a significant burden for laboratory staff. Due to various factors, such as disease prevalence, an increasing population, a rise in the usage of healthcare services, etc., the diagnostic costs continue to rise [11]. Therefore, the question arises as to how to perform a rapid triage of children with suspected COVID-19 prior to qPCR. Artificial intelligence (AI) offers novel and more economical solutions. According to extensive cost–benefit research by Khanna et al. [12], AI has been recognized as a promising and tremendously cost-saving diagnostic tool.

The new technologies of Industry 4.0 have significantly influenced medicine in terms of differential diagnosis, prognosis, and treatment of diseases. Hence, this digitalization and transformation of medicine is also labeled Medicine 4.0. In this era of digitalization in medicine, artificial intelligence has been recognized and used as a promising diagnostic tool and support in the fight against COVID-19 [13,14]. In the largest number of papers published so far, machine-learning algorithms were used to recognize X-ray and/or CT abnormalities in SARS-CoV-2-positive patients [15], while fewer studies focused on clinical laboratory parameters [16]. Most studies included an adult population, while the data for the pediatric population are still insufficient. Additionally, laboratory markers have been mostly used for prognosis rather than preliminary diagnosis and triage of COVID-19 patients [17].

Therefore, the aim of this study was to assess the utility of the most common machine-learning algorithms in the rapid triage of children with suspected COVID-19 using easily accessible and less expensive laboratory parameters.

## 2. Materials and Methods

**Figure 1:**
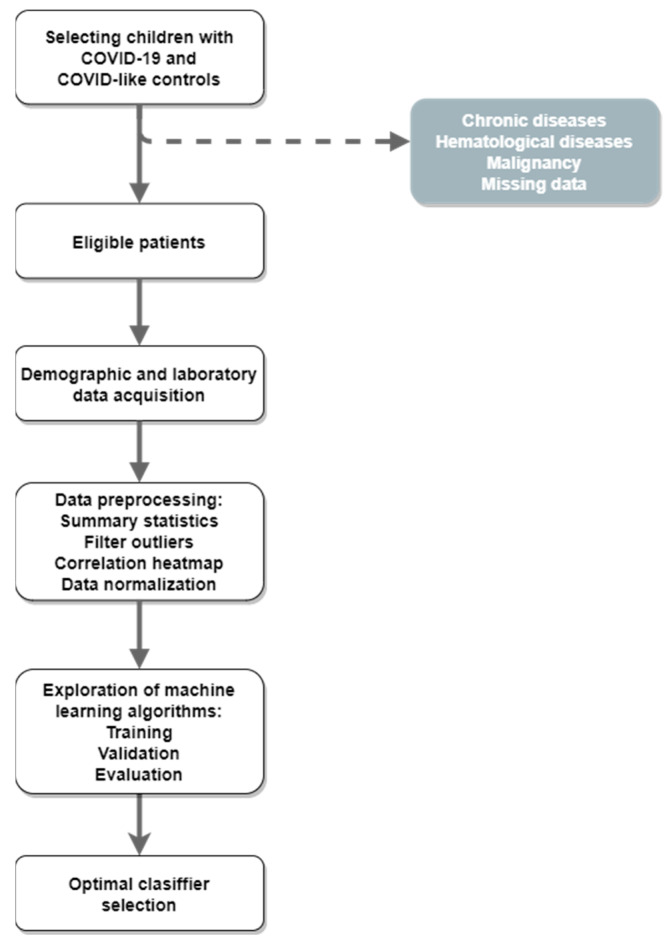
Workflow of predictive modeling.

This cross-sectional study included 280 children with PCR-confirmed SARS-CoV-2 infection (COVID group), treated at the Institute for Children and Youth Health Care of Vojvodina, Novi Sad, Serbia, in the period from March 2020 to December 2022. The control group (non-COVID group) consisted of 286 children with respiratory symptoms who were SARS-CoV-2 PCR-negative. The exclusion criteria were chronic diseases, malignancies, hematological diseases, and missing data (Figure 1). The detection of SARS-CoV-2 antigen in nasopharyngeal swab samples of children was performed by the qPCR technique at the Institute of Public Health of Vojvodina, Novi Sad, Serbia. Children were divided into six age groups according to chronological age: newborn (0–28 days), infant (1–12 months), toddler (1–3 years), preschool (4–6 years), school (7–14 years), and adolescent (15–18 years).

### 2.1. Data Acquisition

Data on parameters of the complete blood count and baseline biochemical parameters, including aspartate aminotransferase (AST), alanine aminotransferase (ALT), gamma-glutamyl transferase (GGT), lactate dehydrogenase (LDH), and C-reactive protein (CRP), were collected on the day of admission. All data were obtained through the institute’s laboratory information system using the structured query language (SQL) code as a searching tool. The blood samples were collected using 0.5 mL violet-topped microtubes with ethylenediaminetetraacetic acid dipotassium salt dehydrate (K2EDTA) as a blood clotting inhibitor (Becton Dickinson, Franklin Lakes, NJ, USA). The values were determined from the hematology analyzer Advia 2120 (Siemens Healthcare, Erlangen, Germany) and chemistry analyzer DxC 700 AU (Beckman Coulter, Brea, CA, USA).

### 2.2. Data Preprocessing

First, all patients with missing data were excluded from the study, following one of the above-mentioned eligibility criteria. Second, the outliers were identified as data points located outside the whiskers of the box plot and excluded from further analysis. Third, Spearman’s correlation was used to screen out highly correlated laboratory parameters in order to minimize the number of input parameters (the threshold value was set to 0.4). A correlation heatmap was used to visualize the strength of relationships between the parameters (Figure S1). Fourth, min–max normalization was applied to transform each parameter into the range [0, 1] in order to treat them with equal weight without distorting the general distribution in the source data.

### 2.3. Baseline Statistical Analyses

Statistical analyses (descriptive and inferential) were performed using open-source software, JASP version 0.16.4.0 (Department of Psychological Methods, University of Amsterdam, Amsterdam, The Netherlands). The significance level for the calculated differences was set at 0.05. For continuous random variables, the normality of distribution was estimated using the Shapiro–Wilk test. Between-group differences were analyzed using the Mann–Whitney U-test. Univariate logistic regression analysis was performed to determine the parameters, which could predict COVID-19 occurrence.

### 2.4. Machine-Learning Algorithms

The following machine-learning algorithms were tested in this study: random forest (RF), support vector machine (SVM), linear discriminant analysis (LDA), artificial neural network (ANN), k-nearest neighbors (KNN), and decision tree (DT). All these algorithms belong to supervised learning, and their goal is classification. The data set was divided into two subsets: the training set and the test set, with an 80:20 split. The training set was validated through stratified cross-validation, where each tuning cycle involved a different non-overlapping holdout data set. The performance of each algorithm was confirmed by additional, independent data set—the test set. The final evaluation of the model included the calculation of accuracy, sensitivity, specificity, positive predictive value, and negative predictive value from the confusion matrix, i.e., the table of predicted and actual values of a classifier. The values were expressed as percentages. Discrimination between groups (COVID and non-COVID) by machine-learning algorithms was presented using receiver operating characteristics (ROC) curves.

### 2.5. Ethical Approval

The study was approved by the Ethics Committee of the Institute for Children and Youth Healthcare of Vojvodina (22 July 2022; No. 3280–2).

## 3. Results

Following the eligibility criteria, in the period from March 2020 to December 2022, a total of 566 children treated at the Institute for Children and Youth Health Care of Vojvodina, Novi Sad, Serbia, were included in the study. The median age of the COVID-19 group was 4.2 years with a female share of 46.4%, while the median age of the non-COVID group was 3.8 years with a female share of 47.9%. Children were divided into six age groups according to chronological age: newborn (7.6%), infant (15.9%), toddler (24.9%), preschool (18.6%), school (17.8%), and adolescent (15.2%).

### 3.1. Clinical Laboratory Features

The initial data included 22 clinical laboratory parameters, a complete blood count, and baseline biochemical parameters. After screening out highly correlated instances (Spearman’s rank correlation coefficient over 0.4), a total of 14 parameters were included in further analysis: white blood cells (WBC), red blood cells (RBC), mean corpuscular volume (MCV), mean corpuscular hemoglobin concentration (MCHC), platelets (PLT), mean platelet volume (MPV), plateletcrit (PCT), platelet distribution width (PDW), absolute lymphocyte count (LYM#), absolute eosinophil count (EOS#), AST, GGT, LDH, and CRP (Table 1). Univariate logistic regression analysis was employed to examine the association of individual laboratory parameters with the presence of SARS-CoV-2 infection in children. The following parameters demonstrated significant diagnostic properties as independent predictors: WBC, MCHC, MPV, and PDW (Table 1). PCR-SARS-CoV-2-negative children had higher values of WBC and MCHC, while children with COVID-19 had higher values of MPV and PDW.

### 3.2. Machine-Learning Algorithm Performances

A comparison of six investigated machine-learning algorithms, based on the standard evaluation metrics, was carried out with a reduced number of instances (Table 2).

**Figure 2:**
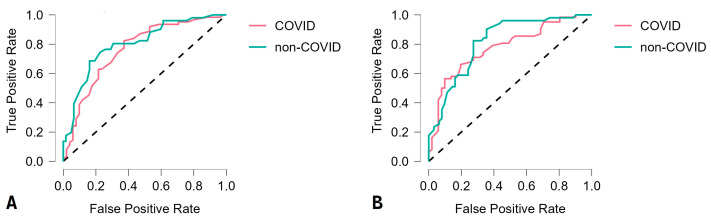
Random forest (**A**) and support vector machine (**B**) receiver operating characteristic curve for the rapid triage of children with suspected COVID-19.

The RF and SVM models demonstrated the highest accuracy of 85% and 82.1%, respectively, while all the other algorithms classified instances with an accuracy lower than 80%. The RF and SVM models demonstrated better sensitivity than specificity and better negative predictive value than positive predictive value. The F1 score, which combines positive predictive value (precision) and sensitivity (recall) using their harmonic means, was higher for the RF than for the SVM model, 85.2% and 82.3%, respectively. After evaluating the performance of the best model (in our study, this was the RF model), the feature importance was compared based on its increase in node purity, i.e., its mean decrease in accuracy. The most prominent instances (node purity over 0.01) were shown to be MPV, WBC, MCHC, PDW, and LYM#. Discrimination between groups (COVID and non-COVID) by machine-learning algorithms was presented using receiver operating characteristics (ROC) curves (Figure 2).

## 4. Discussion

In order to limit the spread of the SARS-CoV-2 virus, it is necessary to detect positive cases as soon as possible and isolate them. However, the small number of available qPCR tests, their high price, and the relatively high percentage of false negative results of these tests brought about the need for additional diagnostic tools [18]. Machine-learning algorithms for automatic disease detection have been increasingly applied in different areas of medicine. Machine learning is a field of artificial intelligence, which provides systems with the ability to automatically learn from experience. The main purpose of these models is to find appropriate patterns in the data, i.e., to produce statistically reliable and reproducible results [19,20].

In this study, the authors evaluated the performance of the six most common machine-learning algorithms: RF, SVM, LDA, ANN, KNN, and DT. The RF and SVM models outperformed the others with an accuracy of 85% and 82.1%, respectively. Both algorithms fall under supervised machine learning. The task of supervised machine-learning algorithms is to “learn” the prediction function h(x) based on a given training data set, so that h(x) is an optimal approximation of the target classes, in this case COVID and non-COVID [20,21].

The SVM machine-learning technique solves the problems of non-linear classification and regression using convex quadratic programming methods. This model only uses instances from the training set that contribute most to the optimal solution of the quadratic programming problem, forming the so-called support vectors. SVM is a very popular and reliable prediction method. During the COVID-19 pandemic, its application has been confirmed for diagnostic purposes [22,23,24], mortality risk assessment [25,26], detecting undertriage in telephone triage [27], etc.

Unlike SVM, which belongs to the category of “individual” algorithms in supervised machine learning, RF belongs to the class of ensemble methods, which combine the results of several individual methods in a certain way. This approach aims to obtain better prediction results than any of the individual methods. For RF construction, an ensemble is formed consisting of several hundred to several thousand DTs. The advantages of DTs, compared to other machine-learning methods, are their simplicity of implementation and the comprehensibility of the procedure. There are rules by which trees are quickly formed, and the output can be easily interpreted. In addition, DTs allow attributes to have missing values, which is not the case with SVM. However, one of the disadvantages of the DT method is its instability. A small change in the input training data can lead to a significant change in the topology of the tree. Instability occurs due to many possible splits, which often have approximately the same importance (competitor splits). Therefore, a small change in the data can lead to a completely different partition, which further introduces changes to all the branches of the tree below it. RF overcomes these limitations by aggregating the prediction results of hundreds of individual trees [21]. Therefore, the RF model has been widely used during the COVID-19 pandemic for disease diagnosis [28,29], predicting patient outcomes [30,31,32], recommending hospitalization [33], processing of healthcare and travel data to identify COVID-infected people [34], etc.

The evaluation metrics for the RF and SVM models based on clinical laboratory data reported in other studies were similar to this study. Our RF model demonstrated an accuracy of 85%, while Çubukçu et al. [35] reported an accuracy of 85.2% in their RF model using complete blood count parameters and clinical chemistry parameters as input variables, but in an adult population. Our SVM model demonstrated an accuracy of 82.1%, thus outperforming a model proposed by Thimoteo et al. [36], who reported an accuracy of 73.7% in their SVM model, including complete blood count parameters only, as well as an adult population.

The model presented in this study cannot outperform the PCR method, which is considered the diagnostic gold standard, with an average efficiency of over 96% [37]. Conversely, the suggested model outperformed the immunochromatography method used in the rapid SARS-CoV-2 antigen tests. In the beginning of the pandemic, only a few antigen tests received emergency use authorization (EUA) from regulatory authorities, indicating their acceptable performance [38]. Subsequently, some of them were reported to have a sensitivity of no more than 30% [39]. Since these tests were widely used for diagnosing active infections, their performance has significantly improved over time. One of the most commonly used rapid antigen tests at our institute has an overall sensitivity of 79.6% [40]. Our RF and SVM models demonstrated a sensitivity of 86% and 84.8%, respectively.

Including additional clinical biochemical analyses in a machine-learning algorithm, such as ferritin [41], fibrinogen, D-dimer [42], procalcitonin, interleukin-6 [43], etc., may increase the accuracy, sensitivity, and specificity of the model. However, minimal blood sample volume is imperative in pediatric health research. For example, a complete blood count analysis can be performed using a total blood volume of 25 µL. Blood volume overdraws in pediatric laboratory medicine should always be taken into consideration from a legal and ethical perspective [44].

Children experience milder symptoms during the course of the SARS-CoV-2 infection in comparison to adult individuals. Age-related differences may reflect disease severity. These age-related differences include differences in immunity, differences in binding affinity of the SARS-CoV-2 target receptors, etc. [45,46]. In our study, children were divided into six age groups according to chronological age: newborn (0–28 days), infant (1–12 months), toddler (1–3 years), preschool (4–6 years), school (7–14 years), and adolescent (15–18 years). According to the systematic review conducted by Carobene et al. [47], only half of the PubMed and Scopus publications on the application of artificial intelligence in COVID-19 diagnostics take demographic data, such as gender and age, into consideration. The reference ranges in pediatric laboratory medicine are strictly defined and age-dependent. Therefore, it is mandatory to compare individuals within the same age group. This is often overlooked by many researchers, which produces misleading conclusions. Another important consideration and strength of the study is the period in which the children were included, i.e., March 2020–December 2022. This period covers several waves of the pandemic, both before and after the vaccination program had started.

Data on laboratory-based machine-learning approaches for the detection and triage of children with COVID-19 are scarce. Previous studies were mainly focused either on the adult population or, in the case of pediatric population, on radiological rather than laboratory findings, and on outcome rather than detection. With that being considered, this study is a unique contribution to pediatric laboratory medicine.

Multiplex PCR and rapid panel antigen tests (lateral immunochromatography) are used in the diagnosis of viral infections in pediatrics. However, the healthcare system faces a constant challenge in finding an additional diagnostic modality, which is fast, reliable, and cheap, such as AI algorithms. Nevertheless, the potential drawbacks of machine learning in personalized laboratory medicine should be taken into consideration. Diagnostics should always strongly rely on human skills, including physical examination, critical perception of medical history, etc. Machine-learning algorithms provide multi-dimensional biomedical data, which should not be overrated and should only be observed as supporting information for making a final diagnosis [48].

Analyzing the cost–benefit properties of the algorithms proposed in this study, it can be concluded that the healthcare system can benefit from these algorithms in terms of both time and money. The turnaround time for SARS-CoV-2 qPCR tests can vary depending on several factors, including testing capacity and demand, laboratory workload and staffing, supply chain issues, transportation, logistics, etc. The average turnaround time for SARS-CoV-2 qPCR tests ranges from a few hours to a couple of days [49]. On the contrary, laboratory tests used as input data in the proposed algorithm can be performed within a few minutes, and the algorithm can deliver results within a few seconds. Taking finances into consideration, the proposed algorithms have an advantage over the SARS-CoV-2 qPCR test. The price for a single SARS-CoV-2 qPCR test may vary depending on several factors, such as the country and healthcare system. However, the average price ranges from around USD 50 to USD 200 per test. On the other hand, the baseline laboratory parameters used in this study can be obtained from automated clinical chemistry analyzers for no more than USD 50 [50,51]. Moreover, all the parameters used in this study are part of routine testing upon admission to our institute. Therefore, the proposed algorithm entails no additional laboratory-related cost. Furthermore, the statistical package used in this study is an open-source program without the need for license-related costs. Based on the above-mentioned claims, it can be inferred that the implementation of the proposed machine-learning algorithms could improve overall testing efficiency with no extra costs for the healthcare facility.

The use of machine learning in COVID diagnostics has several clinical implications, which can greatly impact the detection and management of the disease, such as early detection and diagnosis, improved accuracy and efficiency, risk stratification and prognosis, personalized treatment plans, monitoring disease trends and outbreaks, etc. It is important to note that while machine learning holds great promise, it should be integrated into clinical practice with caution. Rigorous validation, ethical considerations, and ongoing monitoring are necessary to ensure the reliability, safety, and ethical use of machine-learning models in COVID diagnostics [52,53].

There are certain limitations to the approach proposed in this study and some practical considerations for future research to be considered. First, this is a single-center study, which only includes a limited number of children. Second, the pediatric patients in this study are all European. Performing multi-institutional and multi-national studies could evaluate whether the proposed models could perform well in other human races. Third, all children with underlying conditions were excluded from the proposed study, making its clinical applicability for children with co-infections limited. Fourth, only blood counts and baseline biochemical parameters were included in the study, as they are easily and immediately obtained at admission. However, other non-laboratory parameters could also be included, resulting in a potentially better diagnostic model. Fifth, the control group was heterogeneous, without a specific pathogen classification. They were all labeled only as non-COVID, i.e., SARS-CoV-2-negative.

## 5. Conclusions

Herein, the six most common machine-learning algorithms for the rapid triage of children with suspected COVID-19 were presented and validated. The RF and SVM models outperformed the others with fairly high accuracy. Thereby, a set of clinical laboratory features with markedly high prediction potential was identified, including MPV, WBC, MCHC, PDW, and LYM#. The results of this study might have significant clinical applications, helping healthcare providers identify children with COVID-19 in the early stages, prior to PCR and/or antigen testing. Additionally, machine-learning algorithms could improve overall testing efficiency with no extra costs for the healthcare facility. However, the potential drawbacks of machine learning in personalized laboratory medicine should be taken into consideration.

## Figures and Tables

**Table 1:** Laboratory findings as diagnostic markers for children with suspected COVID-19.

Laboratory Parameter ^a^	COVID Group (*n* = 280)	Non-COVID Group (*n* = 286)	Overall (*n* = 566)	*p*-Value	Univariate Analysis		Multivariate Analysis	
					OR (95% CI)	*p*-Value	OR (95% CI)	*p*-Value
**WBC** (10^9^)	7.9 (5.6–11.8)	10.9 (7.9–15.2)	9.4 (6.6–13.9)	**<0.001**	**1.088 (1.055–1.123)**	**<0.001**	**1.052 (1.016–1.089)**	**0.004**
**RBC** (10^12^)	4.5 (4.1–4.9)	4.4 (4.1–4.8)	4.5 (4.1–4.8)	0.359	NA	NA	NA	NA
**MCV** (fL)	80.7 (77.2–85.1)	79.9 (76.1–84.9)	80.1 (76.4–85)	0.130	NA	NA	NA	NA
**MCHC** (g/L)	340 (331–347)	344 (332–352)	342 (331.2–350)	**0.003**	**1.039 (1.024–1.056)**	**<0.001**	**1.029 (1.014–1.044)**	**<0.001**
**PLT** (10^9^)	300 (217–386.2)	342 (239.5–403.5)	315 (230–392.8)	**0.028**	1.001 (0.999–1.003)	0.189	NA	NA
**MPV** (fL)	7.8 (7.1–8.5)	7.4 (6.8–8)	7.5 (7–8.3)	**<0.001**	**1.028 (1.002–1.054)**	**0.031**	1.001 (0.996–1.007)	0.387
**PCT** (%)	0.24 (0.18–0.3)	0.23 (0.18–0.3)	0.23 (0.18–0.3)	0.943	NA	NA	NA	NA
**PDW** (%)	13.8 (11.7–16.3)	12.9 (11.7–14.6)	13.4 (11.7–15.5)	**0.010**	**1.427 (1.283–1.587)**	**<0.001**	**1.183 (1.090–1.283)**	**<0.001**
**LYM**# (10^9^)	2.3 (1.4–3.9)	2.8 (1.9–4.9)	2.6 (1.6–4.4)	**<0.001**	1.054 (0.956–1.161)	0.291	NA	NA
**EOS**# (10^9^)	0.07 (0.03–0.13)	0.1 (0.05–0.15)	0.09 (0.04–0.15)	0.889	NA	NA	NA	NA
**AST** (µkat/L)	0.62 (0.46–0.82)	0.57 (0.44–0.45)	0.58 (0.45–0.79)	**0.048**	0.821 (0.621–1.085)	0.165	NA	NA
**GGT** (µkat/L)	0.24 (0.18–0.47)	0.26 (0.19–0.56)	0.25 (0.18–0.52)	0.124	NA	NA	NA	NA
**LDH** (µkat/L)	4.39 (3.55–5.1)	4.68 (3.73–5.44)	4.4 (3.7–5.3)	**0.017**	1.107 (0.992–1.234)	0.068	NA	NA
**CRP** (mg/L)	5.5 (1.2–30)	13.2 (2.7–71.6)	9.7 (1.6–53.7)	**<0.001**	1.002 (0.999–1.005)	0.171	NA	NA

^a^ Values are median (interquartile range: Q1–Q3); Mann–Whitney U-test. WBC—White blood cells. RBC—Red blood cells. MCV—Mean corpuscular volume. MCHC—Mean corpuscular hemoglobin concentration. PLT—Platelet. MPV—Mean platelet volume. PCT—Plateletcrit. PDW—Platelet distribution width. LYM#—Absolute lymphocyte count. EOS#—Absolute eosinophil count. AST—Aspartate aminotransferase. GGT—Gamma-glutamyl transferase. LDH—Lactate dehydrogenase. CRP—C-reactive protein. Values in bold are statistically significant.

**Table 2:** Machine-learning classifiers for the rapid triage of children with suspected COVID-19.

Classifier	Accuracy (%)	Sensitivity (%)	Specificity (%)	Positive Predictive Value (%)	Negative Predictive Value (%)	F1 Score (%)
Random forest	85.0	86.0	83.9	84.5	85.5	85.2
Support vector machine	82.1	84.8	79.4	80.0	84.4	82.3
Linear discriminant analysis	78.8	81.1	76.7	75.4	82.1	78.1
Neural network	76.1	72.6	80.4	81.8	70.7	76.9
k-nearest neighbors	73.5	71.0	76.5	78.6	68.4	74.6
Decision tree	68.1	65.5	70.7	67.9	68.3	66.7

## Data Availability

Research data are available upon reasonable request.

## Supplementary Materials

The following supporting information can be downloaded at: https://www.mdpi.com/article/10.3390/v15071522/s1, Figure S1: Correlation heatmap.
