# Supplementary figures and images for: Rapid Triage of Children with Suspected COVID-19 Using Laboratory-Based Machine-Learning Algorithms
Source: Viruses. 2023 Jul 8;15(7):1522. doi: 10.3390/v15071522 (PMC10383367; doi:10.3390/v15071522)

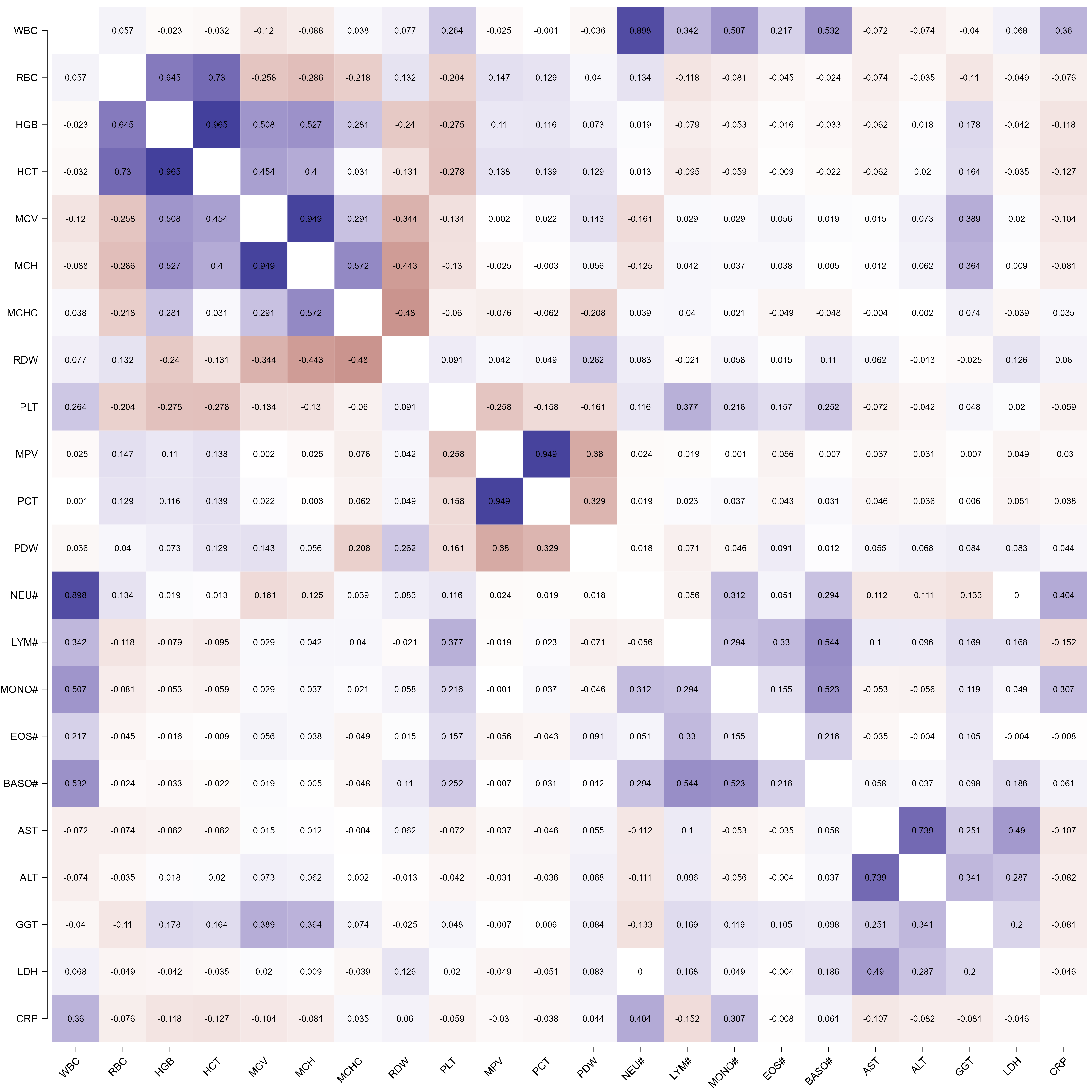

Supplement: Supplementary file 1 [file viruses-15-01522-s001.zip › Figure S1. Correlation heatmap 300dpi-2.png]
